# Efficacy of an Adjuvanted Middle East Respiratory Syndrome Coronavirus Spike Protein Vaccine in Dromedary Camels and Alpacas

**DOI:** 10.3390/v11030212

**Published:** 2019-03-02

**Authors:** Danielle R. Adney, Lingshu Wang, Neeltje van Doremalen, Wei Shi, Yi Zhang, Wing-Pui Kong, Megan R. Miller, Trenton Bushmaker, Dana Scott, Emmie de Wit, Kayvon Modjarrad, Nikolai Petrovsky, Barney S. Graham, Richard A. Bowen, Vincent J. Munster

**Affiliations:** 1 Department of Microbiology, Immunology, and Pathology, Colorado State University, Fort Collins, CO 80521, USA; Danielle.Adney@colostate.edu; 2 Vaccine Research Center, National Institute of Allergy and Infectious Diseases, National Institute of Health, Bethesda, MD 20892, USA; wangling@niaid.nih.gov (L.W.); shiw@mail.nih.gov (W.S.); yi.zhang3@nih.gov (Y.Z.); wkong@mail.nih.gov (W.-P.K.); bgraham@mail.nih.gov (B.S.G.); 3 Laboratory of Virology, Division of Intramural Research, National Institute of Allergy and Infectious Diseases, National Institutes of Health, Hamilton, MT 59840, USA; neeltje.vandoremalen@nih.gov (N.v.D.); megan.r.miller@colostate.edu (M.R.M.); bushmakertj@niaid.nih.gov (T.B.); dana.scott@nih.gov (D.S.); Emmie.deWit@nih.gov (E.d.W.); 4 Military HIV Research, Program, Walter Reed Army Institute of Research, Silver Spring, MD 20910, USA; kmodjarrad@hivresearch.org; 5 Flinders University and Vaxine Pty Ltd, Flinders Medical Centre, Bedford Park, SA 5042, Australia; nikolai.petrovsky@flinders.edu.au

**Keywords:** MERS-CoV, camels, vaccines, One Health

## Abstract

MERS-CoV is present in dromedary camels throughout the Middle East and Africa. Dromedary camels are the primary zoonotic reservoir for human infections. Interruption of the zoonotic transmission chain from camels to humans, therefore, may be an effective strategy to control the ongoing MERS-CoV outbreak. Here we show that vaccination with an adjuvanted MERS-CoV Spike protein subunit vaccine confers complete protection from MERS-CoV disease in alpaca and results in reduced and delayed viral shedding in the upper airways of dromedary camels. Protection in alpaca correlates with high serum neutralizing antibody titers. Lower titers of serum neutralizing antibodies correlate with delayed and significantly reduced shedding in the nasal turbinates of dromedary camels. Together, these data indicate that induction of robust neutralizing humoral immune responses by vaccination of naïve animals reduces shedding that potentially could diminish the risk of zoonotic transmission.

## 1. Introduction

Since the emergence of the Middle East respiratory syndrome coronavirus (MERS-CoV) in 2012, there have been more than 2279 confirmed human cases and 806 deaths, yielding a case fatality ratio of 35% [1,2]. Nearly all cases either have occurred in or been exported from the Middle East. Dromedary camels have been identified as the primary reservoir of MERS-CoV through multiple studies that have employed combinations of diagnostic platforms, to include serology, molecular detection of viral RNA, and virus isolation [3,4,5,6,7,8,9,10,11,12,13,14,15]. Serologic studies suggest that MERS-CoV has been present in dromedary camels for at least three decades in regions as wide ranging as the Middle East, North Africa, and East Africa [16]. The outbreak among human populations has persisted—albeit in low numbers—despite a lack of sustained human-to-human transmission [17,18]. Data from molecular epidemiologic studies suggest that the outbreak has endured through multiple zoonotic spillover events from dromedary camels to humans. Additionally, several studies in Saudi Arabia and Qatar have demonstrated a clear epidemiologic link between camel and human populations [4,19,20,21,22,23,24,25,26].

Although MERS-CoV typically causes severe pneumonia in humans, the virus primarily infects the upper respiratory tract of dromedary camels, from where viable virus is shed in high quantities for approximately 7 days, and where viral RNA can be detected up to 35 days [27]. Occupational exposure that involves close proximity to infected dromedary camels is a major risk factor for human acquisition. This risk profile has been confirmed by studies in Saudi Arabia where camel herders and slaughterhouse workers were much more likely than the general population to either have serologic evidence of past exposure to MERS-CoV or develop MERS [20,26].

The high case fatality rate, continued zoonotic transmission, and sustained animal reservoir of MERS-CoV underscore the need for intervention strategies focused on preventing zoonotic transmission. Here, we evaluated the efficacy of an intramuscular, adjuvanted S1 subunit vaccine to prevent infection or reduce shedding in dromedary camels (*Camelus dromedarius*) and alpaca (*Vicugna pacos*).

## 2. Materials and Methods

### 2.1. Vaccine Development

**Figure 1:**
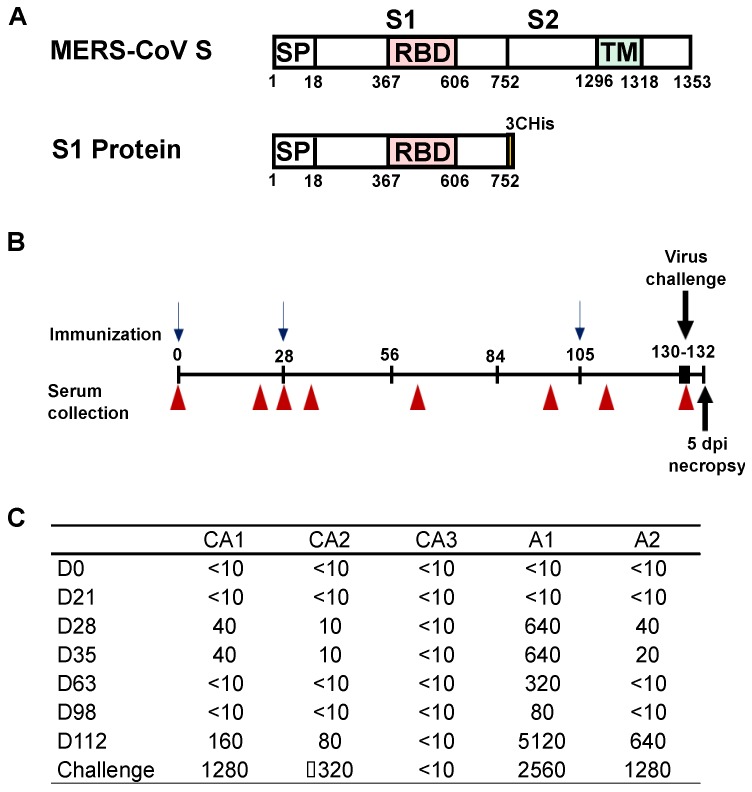
Vaccine immunogens and neutralizing antibody responses. (**A**) Schematic representation of the full MERS-CoV spike protein (top) and the S1 subunit used as antigen for vaccination (bottom). (**B**) Schematic overview of the immunization timeline with the immunization (blue arrows), serum sampling (red triangle), and challenge and euthanasia (black arrows) time points indicated. (**C**) Neutralizing antibody titers in dromedary camels and alpacas vaccinated with adjuvanted MERS-CoV S1 as determined by PRNT (90% neutralization).

DNA expression vectors were produced encoding the S1 portion of the Spike glycoprotein of MERS-CoV England1 strain (strain England1, GenBank ID: AFY13307) (Figure 1). Sequences were reverse-translated and codon-optimized for human cell expression as described [28]. Proteins were produced by transfecting Expi293 cells with the mammalian expression vector VRC8400 expressing the codon-optimized S1 gene. Protein was purified from transfected cell culture supernatants with HisTrap HP Hiload 16/60 Superdex columns (GE Healthcare, Piscataway, NJ, USA), and stored at −80 °C in PBS until use.

### 2.2. Ethics Statement

All experiments were approved by the Colorado State University Institutional Animal Care and Use Committee. Work with infectious MERS-CoV strains under BSL3 conditions was approved by the Institutional Biosafety Committee (IBC). Inactivation and removal of samples from high containment was performed according to IBC-approved standards.

### 2.3. Study Design

To evaluate the effect of vaccination on MERS-CoV shedding, we used both the actual animal reservoir (camels) and a surrogate infection model (alpaca) [27,29]. Five MERS-CoV seronegative dromedary camels and four alpaca were purchased by private sale for use in this study. All animals were born in the United States and were all adult intact males for the Alpaca; one intact adult male, one castrated adult male, and one adult female for the vaccinated dromedary camels; and three intact male dromedary camels for the unvaccinated control animals. Animals were fed ad libitum, housed in outdoor pens during immunization and moved into an Animal Biosafety Level 3 facility one week prior to challenge to allow for acclimation to the facility.

Vaccinated animals were given 400 μg S1 protein combined with 40 mg Advax HCXL adjuvant (a delta inulin formulation, Vaxine Pty Ltd., Bedford Park, SA, Australia), in two 1 mL intramuscular injections given on each shoulder on day 0 and day 28, and then given 400 μg S1 protein in the Sigma Adjuvant System (an oil-in-water emulsion, Sigma-Aldrich, St. Louis, MO, USA) on day 105. The Advax adjuvant was chosen for stimulating broad immunogenicity in dromedary camels [30], the Sigma Adjuvant System for known induction of MERS-CoV neutralizing antibodies [28].

Animals were challenged with MERS-CoV strain HCoV/EMC/2012 in three groups along a staggered schedule, due to facility limitations. Group 1 was inoculated 130 days after first vaccination and consisted of vaccinated camel 3 (CA3), group 2 was inoculated 131 days after first vaccination and consisted of unvaccinated camels 4 and 5 (CA4 and CA5) and vaccinated alpaca 1 and 2 (A1 and A2), and group 3 was inoculated 132 days after first vaccination and consisted of vaccinated camels 1 and 2 (CA1 and CA2) and unvaccinated alpaca 3 and 4 (A3 and A4). Animals were sedated with xylazine and then inoculated intranasally with a total dose of 10^7^ TCID_50_ of a human isolate of MERS-CoV (strain HCoV-EMC/2012) as described previously [27]. Camels were inoculated with 5 mL per nare, and alpaca were inoculated with 3 mL per nare. All animals were evaluated at least once daily for temperature, nasal discharge, activity level, and food consumption. Nasal swabs were collected daily and placed immediately into viral transport medium or virus lysis buffer and then frozen until processing. Animals were sedated on day 5 post-inoculation and humanely euthanized with intravenous pentobarbital. The following tissues were collected for viral isolation and formalin-fixed for immunohistochemistry and histopathology: nasal turbinates, larynx, trachea, lung (right apical, left apical, right caudal, left caudal lobes), kidney, spleen, mediastinal and mesenteric lymph nodes and muscle. Historical samples from a previously infected camel euthanized on day 5 post inoculation [27] were included as an additional control (CA6). CA6 in the present study corresponds to camel 1 in the publication by Adney et al. [27].

### 2.4. Immunization and Serology

Blood was collected on day 0, 21, 28, 35, 63, 98, 112 after first vaccination and at time of challenge (day 130–132) into serum-separating tubes. Serum was analyzed for MERS-CoV neutralization activity by plaque reduction neutralization test (PRNT) as described previously using a 90% neutralization cutoff [27].

### 2.5. Virus Titration

Nasal swabs and tissue samples were titrated by plaque assay in Vero E6 cells. In short, homogenized tissue was titrated in BA-1 medium and plaques were counted on both days 1 and 3 following the second overlay as described previously [27].

### 2.6. RNA Extraction and qRT-PCR

RNA was extracted from swabs, urine and fecal samples using the QiaAmp Viral RNA kit (Qiagen). For detection of viral RNA, 5 μL of RNA was used in a one-step real-time RT-PCR upE assay using the Rotor-GeneTM probe kit (Qiagen) as described previously. Standard dilutions of a titered virus stock were run in parallel, to calculate TCID_50_ equivalents in the samples.

### 2.7. MERS-CoV Spike Glycoprotein Sequencing

Total RNA from nasal turbinate tissue samples from dromedary camels and alpaca were extracted using the RNeasy Mini Kit (Qiagen) and cDNAs were synthesized using random hexamers and the High Capacity RNA to cDNA Kit (Thermo Fisher, Waltham, MA, USA). cDNA was subsequently used to PCR-amplify the MERS-CoV S using iProof High-Fidelity DNA Polymerase (Biorad, Hercules, CA, USA) according to the manufacturer’s protocol; primer sequences are available upon request. Sequences were assembled on SeqMan Pro (DNASTAR, Madison, WI, USA) and analyzed on MegAlign (DNASTAR, Madison, WI, USA) by comparison to the MERS-CoV (strain HCoV-EMC/2012) input sequence.

### 2.8. Histopathology and Immunohistochemistry

Dromedary camel and alpaca tissues were evaluated for pathology and presence of viral antigens. Tissues were fixed for >7 days in 10% neutral-buffered formalin and placed in cassettes and processed with a Sakura VIP-5 Tissue Tek, on a 12-hour automated schedule, using a graded series of ethanol, xylene, and ParaPlast Extra. Embedded tissues are sectioned at 5 μm and dried overnight at 42 degrees C prior to staining. All tissues were processed for immunohistochemistry using the Discovery XT automated processor (Ventana Medical Systems) with a DABMap (Ventana Medical Systems) kit. To detect MERS-CoV antigen, immunohistochemistry was performed using a rabbit polyclonal antiserum against HCoV-EMC/2012 (1:1000) as a primary antibody as described previously [27]. Grading of histopathology and immunohistochemistry was done blinded by a board-certified veterinary pathologist.

### 2.9. Statistical Analysis

*p* values were calculated in a 2-way ANOVA with Sidak’s multiple comparisons test using the Prism software (Version 6.04, GraphPad, La Jolla, CA, USA). Statistically significant differences met a threshold (α) of 0.05.

## 3. Results

### 3.1. Humoral Responses in Dromedary Camels and Alpaca Vaccinated against MERS-CoV

Three dromedary camels (CA1, CA2, CA3) and two alpaca (A1, A2) were vaccinated with an adjuvanted S1-protein subunit vaccine. Three camels (CA4, CA5, CA6) served as unvaccinated controls; CA6 is a historical control [27]. Two alpaca (A3, A4) served as unvaccinated controls for the alpaca group. Animals were vaccinated on days 0 and 28 with 400 μg of S1 protein (Figure 1A) co-formulated with 40 mg Advax^TM^ HCXL adjuvant (Vaxine Pty Ltd, Adelaide, Australia) [30]. The admixed product was delivered at each time point as two 1 mL intramuscular injections in each shoulder. All animals were boosted on day 105 with 400 μg of S1 protein emulsified in Sigma Adjuvant System (Sigma Aldrich Co. LLC. St. Louis, MO, USA) to complete a 0, 4-, and 15-week immunization schedule (Figure 1B). Serum from vaccinated animals was collected and evaluated by plaque reduction neutralization test (PRNT) with MERS-CoV strain HCoV-EMC/2012 (Figure 1C). On day 28 after priming vaccination, low levels of MERS-CoV neutralizing antibodies were detected in two of three the camels (virus neutralizing titers of 1:40, 1:10, and <10 respectively; Figure 1C). The day 28 boost did not result in an increase in neutralizing titers in the PRNT assay and neutralizing titers decreased between the second and third boost. The third immunization resulted in a quick increase in neutralizing titer in the two camels that responded to the vaccine; neutralizing titers were high in these two camels by the time of challenge. Neutralizing antibodies were not detected in CA3 (<1:10) at any point during the experiment (Figure 1C). A stronger neutralizing response was observed in the alpaca after vaccination, with virus neutralizing titers of 1:640 and 1:40 four weeks after the initial vaccination to end titers of 1:2560 and 1:640 at the time of challenge (Figure 1C).

### 3.2. Vaccine Efficacy in Preventing MERS-CoV Disease and Virus Shedding

Animals were challenged intranasally with 10^7^ 50% tissue culture infectious dose (TCID_50_) of MERS-CoV (strain HCoV-EMC/2012) and euthanized and necropsied at 5 days post-inoculation (dpi). Only the unvaccinated control camels developed mild clinical disease. Nasal discharge was observed in concurrent control camels at 2 dpi, but quickly resolved. Observable nasal discharge was not detected in the vaccinated camels nor in any of the alpaca. Minor temperature fluctuations were detected in several of the camels and alpaca; however, there was no appreciable fever associated with infection (Figure S1).

**Figure 2:**
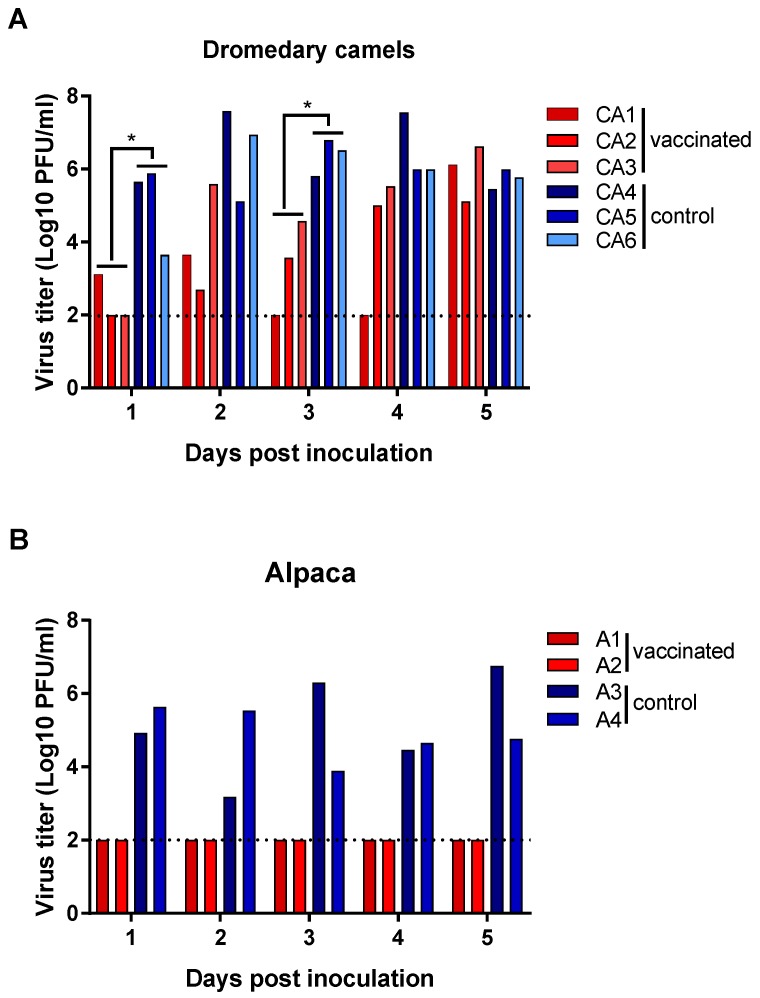
Virus shedding in nasal swabs of vaccinated and unvaccinated dromedary camels and alpaca after challenge with MERS-CoV. Nasal swabs were obtained from dromedary camels (**A**) and alpaca (**B**) daily after MERS-CoV challenge and virus titers in swabs were determined in a plaque assay. Red bars indicate vaccinated animals and blue bars indicate unvaccinated control animals. A dashed line indicates the detection limit of the assay. Asterisks indicate statistically significant difference between geometric mean titers (*p* < 0.05).

Virus shedding was first detected in nasal swabs from unvaccinated control camels at 1 dpi and every day thereafter until euthanasia at 5 dpi (Figure 2A). Despite the presence of neutralizing antibodies in two of the three vaccinated camels, all animals shed virus after challenge. The two vaccinated camels with a detectable humoral immune response (CA1 and CA2) exhibited reduced viral shedding through 4 dpi. At 5 dpi, there were no differences in MERS-CoV titers measured in nasal swabs between vaccinated and control camels. The vaccinated camel without detectable neutralizing antibody titers (CA3) did not shed virus on 1 dpi but otherwise exhibited virus shedding kinetics that were similar to those of unvaccinated controls. Overall, infectious MERS-CoV titers in nasal swabs differed statistically significantly between vaccinated and unvaccinated camels on 1 and 3 dpi (*p* < 0.05). In contrast to the camels, vaccination of alpaca resulted in complete protection from MERS-CoV shedding. No infectious virus was detected in the nasal swabs collected from the vaccinated alpaca (A1, A2), while infectious virus was detected in unvaccinated alpaca every day until necropsy at 5 dpi (Figure 2B).

**Figure 3:**
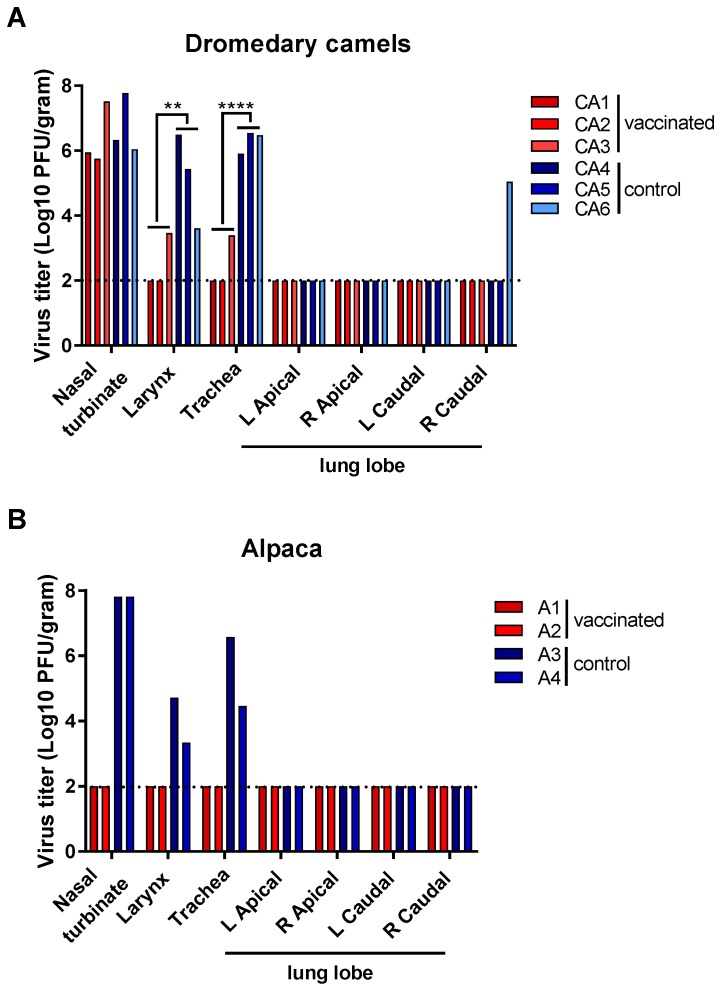
Infectious virus in respiratory tissues of vaccinated and unvaccinated dromedary camels and alpaca after challenge with MERS-CoV. Dromedary camels (**A**) and alpaca (**B**) were euthanized on 5 dpi, tissues were collected, and virus titers were determined in a plaque assay. Red bars indicate vaccinated animals and blue bars indicate unvaccinated control animals. A dashed line indicates the detection limit of the assay. Asterisks indicate statistically significant difference between geometric mean titers (** *p* < 0.01; **** *p* < 0.0001). L: left; R: right.

Infectious virus was detected in the nasal turbinates, larynx, and trachea of all unvaccinated camels (Figure 3A), confirming that the virus stock prepared for challenge resulted in productive infection. Additionally, infectious virus was detected in 1 of 4 lung lobes of CA6. All three vaccinated camels had infectious virus in their nasal turbinates at titers similar to those of the unvaccinated camels. However, virus titers in the larynx and trachea were significantly lower in the vaccinated camels than in unvaccinated controls (Figure 3A). Viral RNA was also detected outside the respiratory tract of camels, predominantly in the lymph nodes (Figure S2A).

Both unvaccinated alpaca had high titers of infectious virus present in their nasal turbinates and lower titers in the larynx and trachea. Neither infectious virus nor viral RNA could be detected in the respiratory tract tissues of the vaccinated alpaca (Figure 3B and Figure S2B). In the unvaccinated control alpaca, low amounts of viral genomic RNA were detected outside the respiratory tract (Figure S2B).

### 3.3. Histopathological Changes in Vaccinated Versus Control Animals

**Figure 4:**
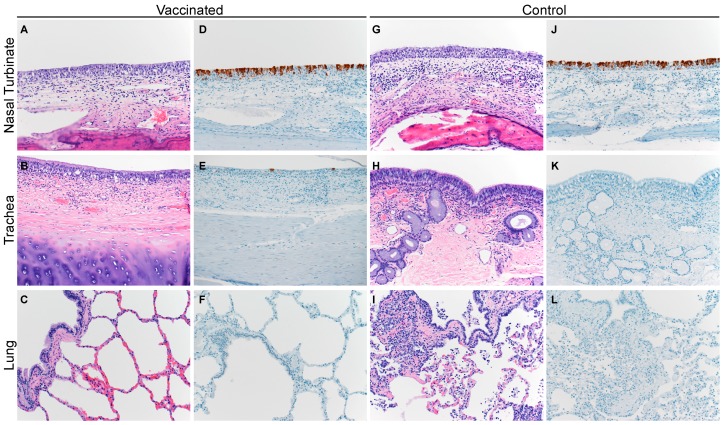
Histopathological changes in the respiratory tract of vaccinated and unvaccinated dromedary camels after challenge with MERS-CoV. Nasal turbinate, trachea, and lung were collected from vaccinated (**A**–**F**; CA3 shown) and unvaccinated control animals (**G**–**L**; CA5 shown) on 5 dpi and stained with hematoxylin and eosin (**A**–**C** and **G**–**I**), or a polyclonal anti-MERS-CoV antibody panels (**D**–**F** and **J**–**L**). MERS-CoV antigen is visible as a red brown staining in the immunohistochemistry panel. Vaccinated and unvaccinated camel groups displayed multifocal, minimal-to-mild, acute rhinitis. MERS-CoV antigen was primarily detected in the nasal turbinates. Magnification: 400×.

All camels displayed, on histologic section, a multifocal, minimal-to-mild, acute rhinitis (Figure 4A,G). The nasal turbinates exhibited a loss of cilia and cellular polarity, occasional necrotic epithelial cells admixed with small numbers of neutrophils, and rare erosions of respiratory epithelium. The underlying submucosa was infiltrated by small-to-moderate numbers of lymphocytes and plasma cells with fewer macrophages and neutrophils. The non-responsive vaccinated camel, CA3, and control CA4 displayed a minimal-to-mild multifocal acute tracheitis, as evidenced by a loss of individual epithelial cells and cell polarity. Necrosis and infiltration of the epithelium by small numbers of neutrophils were observed. None of the camels showed pathological changes in the lower respiratory tract (Figure 4B,C,H,I). Immunohistochemistry (IHC) demonstrated the presence of MERS-CoV antigen in the epithelial cells of the nasal turbinates of CA1-5 (Figure 4D,J). Viral antigen was also detected in the tracheal epithelial cells of CA3 and CA4, but not in CA1 or CA2 (Figure 4E).

**Figure 5:**
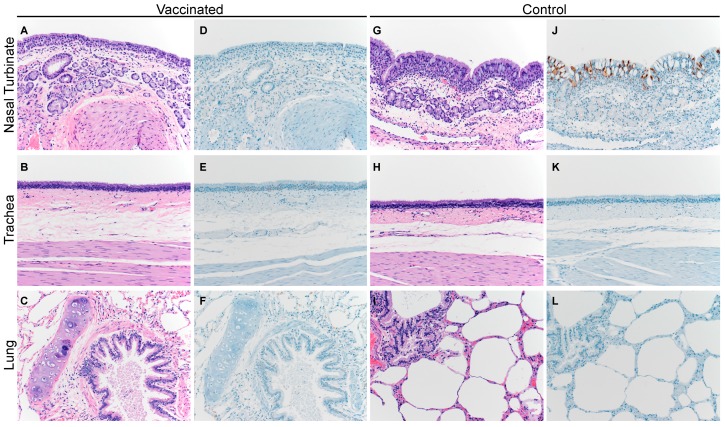
Histopathological changes in the respiratory tract of vaccinated and unvaccinated alpaca after challenge with MERS-CoV. Nasal turbinate, trachea, and lung were collected from vaccinated (**A**–**F**; A1 shown) and unvaccinated control animals (**G**–**L**; A3 shown) on 5 dpi and stained with hematoxylin and eosin (**A**–**C** and **G**–**I**), or a polyclonal anti-MERS-CoV antibody panels (**D**–**F** and **J**–**L**). MERS-CoV antigen is visible as a red brown staining in the immunohistochemistry panel. No histopathological changes were observed in the vaccinated alpaca whereas the unvaccinated control alpaca displayed multifocal, minimal-to-mild, acute rhinitis. MERS-CoV antigen was only detected in the nasal turbinates. Magnification: 400×.

Similar to the camels, the unvaccinated alpaca displayed a multifocal, minimal-to-mild, acute rhinitis (Figure 5G) with loss of cilia and cellular polarity, occasional necrotic epithelial cells admixed with small numbers of neutrophils, and rare erosions of respiratory epithelium. The subjacent submucosa was infiltrated by small-to-moderate numbers of lymphocytes and plasma cells with fewer macrophages and neutrophils. IHC confirmed the presence of MERS-CoV antigen within the epithelial cells of the nasal turbinates of these control alpaca (Figure 5J). No histopathological changes or MERS-CoV antigen were detected in any of the vaccinated alpaca (Figure 5G–L).

## 4. Discussion

Given the importance of the zoonotic reservoir of MERS-CoV in sustaining the current human epidemic, strategies to interrupt the transmission from dromedary camels to humans may serve as an effective countermeasure [31]. This strategy has been implemented in the setting of Hendra virus infection in Australia, where vaccination of horses is aimed at interrupting the transmission chain of Hendra virus from fruit bats to horses to humans [32]. Likewise, dromedary camels are the drivers of zoonotic transmission of MERS-CoV and vaccination of dromedary camels to reduce shedding of MERS-CoV would likely limit its potential for zoonotic transmission.

Large fractions of dromedary camels in the Middle East and North and East Africa have antibodies specific to MERS-CoV [3,6,8,9,12,14,33,34]. The degree of protection from reinfection provided by these antibodies is currently unclear. Evidence points to the ability of MERS-CoV to re-infect dromedary camels despite the presence of antibodies in serum, as MERS-CoV seropositive camels that still shed detectable MERS-CoV from the respiratory tract have been found in the Middle East [8]. However, a correlation between strong humoral responses in seropositive adult dromedary camels and low prevalence of MERS-CoV was observed in Jordan, whereas seropositive young camels <3 years old with weaker humoral responses displayed high MERS-CoV prevalence [34], suggesting that antibodies against MERS-CoV afford some protection from reinfection or virus shedding. This observation also suggests that younger animals are the main driving force behind the circulation of MERS-CoV and should be the primary target for vaccination strategies.

This study highlights several important issues to consider when developing a dromedary camel vaccine against MERS-CoV. First of all, virus shedding was reduced, but not completely absent in the presence of antibodies in dromedary camels after vaccination. However, virus replication in vaccinated animals was largely confined to the nasal turbinates, whereas the MERS-CoV replication in unvaccinated animals extended deeper down the respiratory tract into the trachea. In a study using intranasal administration of a modified vaccinia virus Ankara virus vector vaccine expressing full length Spike protein, delayed shedding was also observed in one of the vaccinated dromedary camels [35]. After vaccination, low neutralizing antibody titers were detected in the upper respiratory tract. The apparent low neutralizing capacity of the upper respiratory tract combined with continuous exposure to MERS-CoV (from co-housed animals) could potentially deplete the neutralizing capacity by scavenging the available neutralizing antibodies until the neutralizing capacity is sufficiently reduced to allow for virus replication. Therefore, vaccine platforms aimed specifically at inducing high levels of mucosal immunity or those that induce humoral as well as cellular immunity may be required for complete protection. In-depth studies to determine the correlates of protection in dromedary camels would aid the development of better-targeted vaccines, especially the correlation of levels of neutralizing antibodies in the serum vs. protection against MERS-CoV in the upper respiratory tract. Second, the response to vaccination in dromedary camels varies widely, as one out of three camels in the present study did not respond to vaccination at all. This camel was a ten-year-old male, housed in a pen adjacent to a mature female. Most of the vaccination schedule coincided with the mating period of this camel. Thus, it is possible, though unclear, that reproductive status impacted the camel’s immune response. Variable neutralizing responses after MERS-CoV vaccination have been reported in a previous dromedary camel vaccine study. One animal in a group of three failed to mount a detectable neutralizing response after vaccination with a DNA vaccine expressing full length Spike protein [36]. Vaccine development efforts should thus focus on vaccine platforms that uniformly induce immunity in dromedary camels. Finally, intranasal challenge with MERS-CoV yielded distinctly different outcomes in the alpaca and dromedary camels. Upon viral challenge, both immunized alpaca were completely protected from MERS-CoV infection. Thus, although alpaca are an attractive alternative experimental model to dromedary camels since MERS-CoV replication dynamics in alpaca appear to be similar to those in dromedary camels [29,37] but alpaca are easier to obtain and house, vaccine efficacy ultimately has to be confirmed in the dromedary camel model.

## Supplementary Materials

The following are available online at https://www.mdpi.com/1999-4915/11/3/212/s1.
