# Supplementary material for: Efficacy of an Adjuvanted Middle East Respiratory Syndrome Coronavirus Spike Protein Vaccine in Dromedary Camels and Alpacas
Source: Viruses. 2019 Mar 2;11(3):212. doi: 10.3390/v11030212 (PMC6466352; doi:10.3390/v11030212)
Supplement: Supplementary file 1 [file viruses-11-00212-s001.pdf]

Supplemental information

Efficacy of an Adjuvanted Middle East Respiratory Syndrome Coronavirus Spike Protein Vaccine in Dromedary Camels and Alpacas

Danielle R. Adney, Lingshu Wang, Neeltje van Doremalen, Wei Shi, Yi Zhang, Wing-Pui Kong, Megan R. Miller, Trenton Bushmaker, Dana Scott, Emmie de Wit, Kayvon Modjarrad, Nikolai Petrovsky, Barney S. Graham, Richard A. Bowen, Vincent J. Munster

A

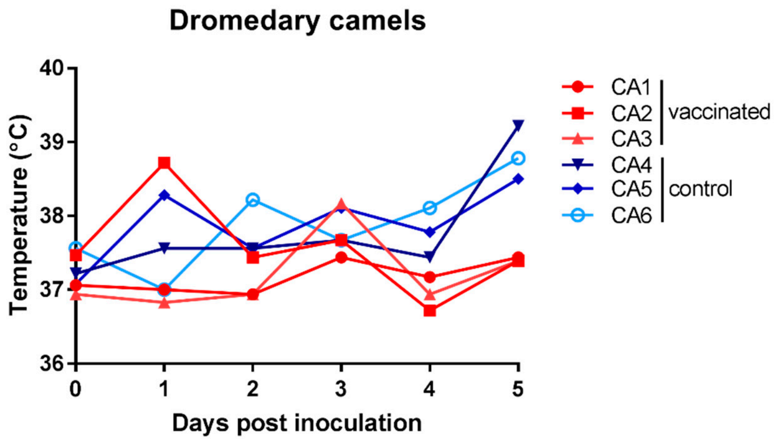

B

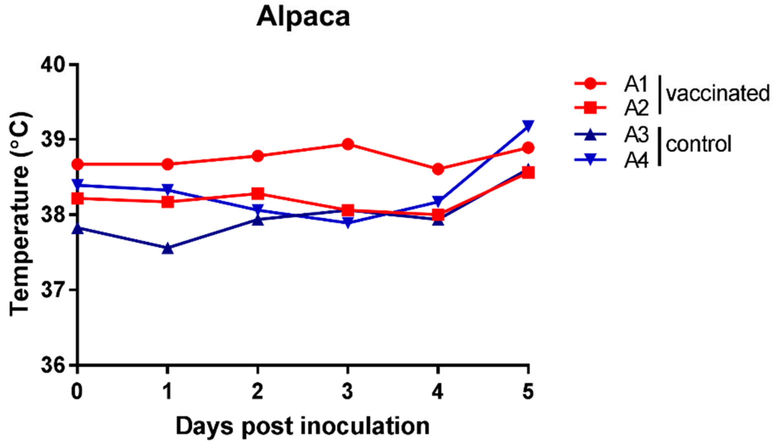

Figure S1. Changes in body temperature of vaccinated and unvaccinated dromedary camels and alpaca after challenge with MERS-CoV. Rectal temperatures of dromedary camels (A) and alpaca (B) were measured daily after challenge with MERS-CoV. Red symbols and lines indicate vaccinated animals, blue symbols and lines indicate unvaccinated control animals.

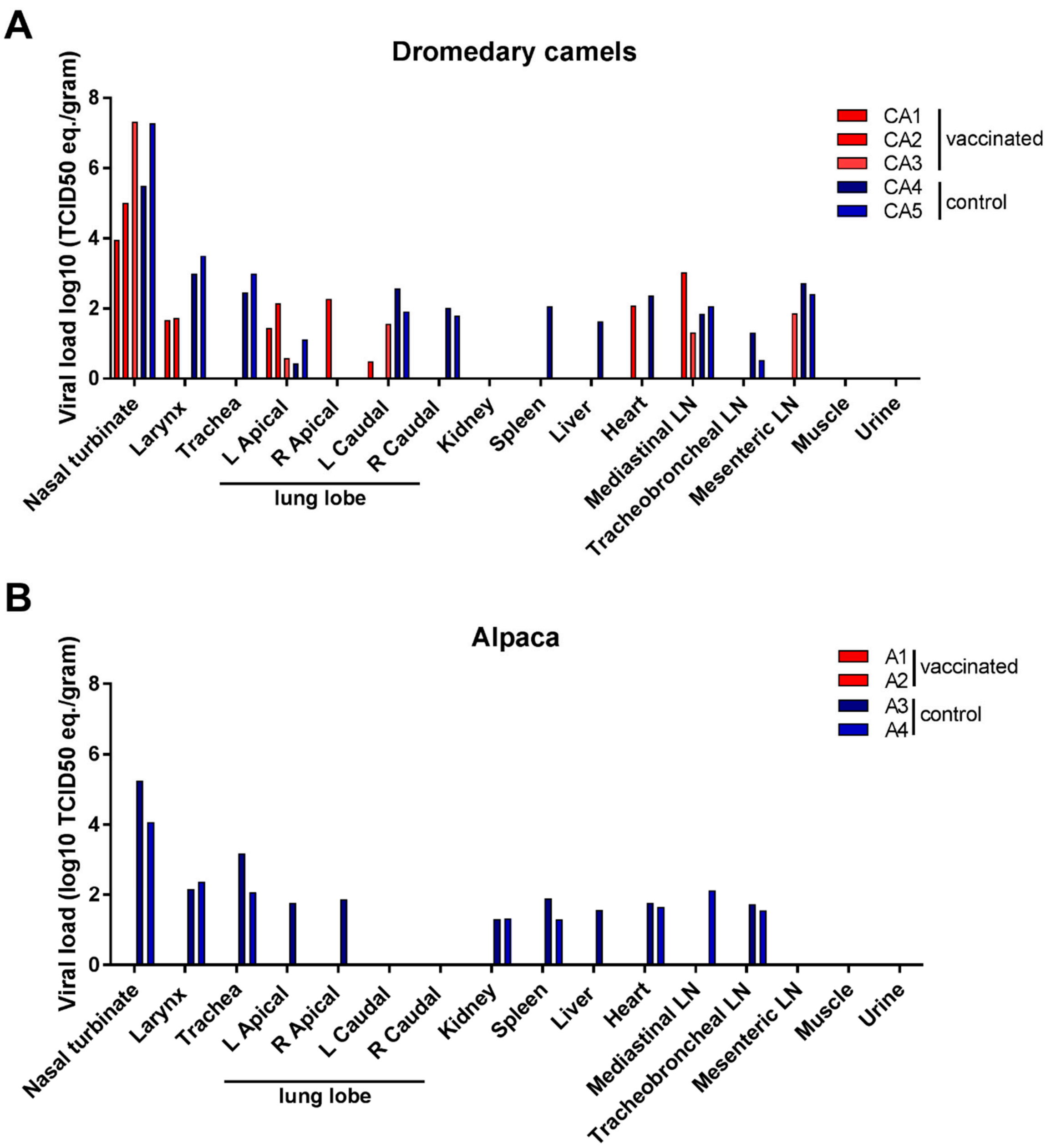

Figure S2. Viral RNA in tissues of vaccinated and unvaccinated dromedary camels and alpaca after challenge with MERS-CoV. Dromedary camels (A) and alpaca (B) were euthanized on 5 dpi, tissues were collected, RNA was extracted and viral load was determined as TCID50 equivalents per gram tissue by qRT-PCR Red bars indicate vaccinated animals and blue bars indicate unvaccinated control animals. L: left; R: right; LN: lymph node.
